# The need for logbooks to evolve in the undergraduate medical setting

**DOI:** 10.1007/s40037-015-0249-x

**Published:** 2016-01-07

**Authors:** Pishoy Gouda

**Affiliations:** University of Alberta, Edmonton, Canada

Logbooks and reflection are an integral part of medical education at all levels of the medical profession. In the undergraduate setting, the ideal logbook is a tool that guides medical students through their clinical rotation by highlighting important clinical objectives, promoting self-reflection and providing an opportunity to obtain feedback from preceptors [1]. In addition, accurate logbook data can be used by training programmes to identify gaps in the curriculum and improve the learning experience for students. Unfortunately logbooks are frequently inappropriately designed, resulting in their suboptimal use by students and institutions alike.

Often logbooks are used as a surrogate marker to measuring student attendance, resulting in both student and staff frustration. A single student evaluation requiring the consultant physician to confirm an acceptable attendance rate, in conjunction with sign in sheets at tutorials, lectures and other core activities, is likely to provide the same incentive for students to attend clinical attachments. This alleviates the need for multiple signatures to confirm attendance at clinics, ward rounds, theatre, etc. Requesting staff signatures to confirm patient interactions does little other than ensure that students document encounters; studies have shown that very few student logbook entries are ever rejected or questioned by the supervising physician [2].

It should be noted that students value the guidance provided by logbooks, [3] indicating approximately how many patients they should be seeing, what clinical skills they need to develop and what procedures they should be attending. However, overburdening students with a myriad of signatures to collect diminishes any potential for a meaningful reflective opportunity, which is already difficult to encourage [4]. While logbooks cannot be used as a substitute for appropriate feedback from supervising physicians, when appropriately designed and used, logbooks can be used as a tool by supervisors to provide thoughtful and meaningful feedback to trainees.

The design and use of logbooks in the undergraduate medical setting needs to evolve into a tool that highlights the importance of quality rather than quantity of patient interactions, encourages a deeper level of reflection and provides the opportunity to obtain meaningful feedback from clinical supervision. Medicine is a clinical art. Medical students need to appreciate the importance of seeing, experiencing and practising this art in the clinical setting; the logbook should support this concept.
